# “Within a minute” detection of focal cortical dysplasia

**DOI:** 10.1007/s00234-021-02823-7

**Published:** 2021-10-09

**Authors:** Horst Urbach, Marcel Heers, Dirk-Matthias Altenmueller, Andreas Schulze-Bonhage, Anke Maren Staack, Thomas Bast, Marco Reisert, Ralf Schwarzwald, Christoph P. Kaller, Hans-Juergen Huppertz, Theo Demerath

**Affiliations:** 1 Department of Neuroradiology, Medical Center, University of Freiburg, Breisacher Str. 64, 79106 Freiburg, Germany; 2 Faculty of Medicine, University of Freiburg, Freiburg, Germany; 3 Department of Epileptology, Medical Center, University of Freiburg, Freiburg, Germany; 4 Epilepsy Centre, Kehl-Kork, Germany; 5 Department of Medical Physics, Medical Center, University of Freiburg, Freiburg, Germany; 6 Swiss Epilepsy Centre, Klinik Lengg AG, Zurich, Switzerland

**Keywords:** Epilepsy, Focal cortical dysplasia, Postprocessing, MP2RAGE

## Abstract

**Purpose:**

To evaluate a MRI postprocessing tool for the enhanced and rapid detection of focal cortical dysplasia (FCD).

**Methods:**

MP2RAGE sequences of 40 consecutive, so far MRI-negative patients and of 32 healthy controls were morphometrically analyzed to highlight typical FCD features. The resulting morphometric maps served as input for an artificial neural network generating a FCD probability map. The FCD probability map was inversely normalized, co-registered to the MPRAGE2 sequence, and re-transferred into the PACS system. Co-registered images were scrolled through “within a minute” to determine whether a FCD was present or not.

**Results:**

Fifteen FCD, three subcortical band heterotopias (SBH), and one periventricular nodular heterotopia were identified. Of those, four FCD and one SBH were only detected by MRI postprocessing while one FCD and one focal polymicrogryia were missed, respectively. False-positive results occurred in 21 patients and 22 healthy controls. However, true positive cluster volumes were significantly larger than volumes of false-positive clusters (*p* < 0.001). The area under the curve of the receiver operating curve was 0.851 with a cut-off volume of 0.05 ml best indicating a FCD.

**Conclusion:**

Automated MRI postprocessing and presentation of co-registered output maps in the PACS allowed for rapid (i.e., “within a minute”) identification of FCDs in our clinical setting. The presence of false-positive findings currently requires a careful comparison of postprocessing results with conventional MR images but may be reduced in the future using a neural network better adapted to MP2RAGE images.

## Introduction

Malformations of cortical development comprise heterogeneous disorders of disrupted cerebral cortex formation caused by various genetic, infectious, vascular, or metabolic etiologies [1]. Among those, focal malformations represent an important subgroup as they may be amenable to epilepsy surgery. Focal malformations include focal cortical dysplasia (FCD) but also gray matter heterotopia and focal polymicrogyria.

Gray matter heterotopia are clusters of normal neurons in abnormal locations and commonly categorized into periventricular nodular heterotopia (previously designated as subependymal heterotopia), subcortical heterotopia, and subcortical band heterotopia (SBH) (previously called double cortex) [1]. Polymicrogyria means an excessive number of abnormally small cerebral gyri, most commonly in a bilateral location in the posterior parts of the Sylvian fissures. However, any part of the cerebral cortex including the frontal, occipital, and temporal lobes can be affected [1].

**Fig. 1:**
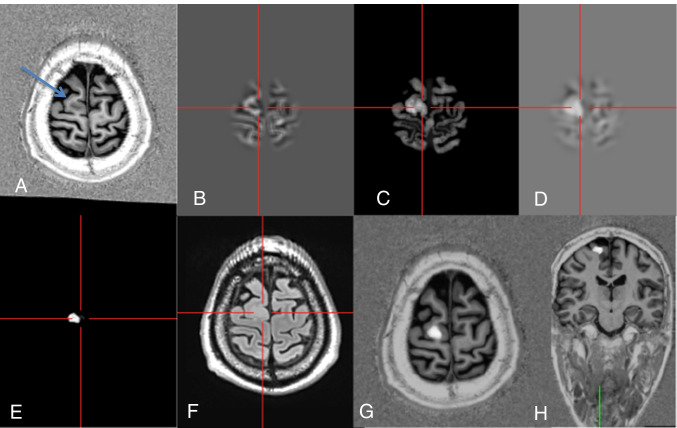
Axial MP2RAGE image with a FCD IIB of the right superior frontal gyrus (**A**: arrow). Postprocessing with normalization, segmentation, and subtraction/division from a database with 154 healthy controls results in the calculation of junction (**B**), thickness (**C**), and extension images (**D**). These serve among others as input maps for an ANN that creates binary output maps in which the lesion is displayed in gray tones (**E**). An axial FLAIR image helps to separate the FCD and false positives (**F**). At the end, co-registered output and MP2RAGE maps are inversely normalized and sent back to the PACS system, in which they are viewed by scrolling through the co-registered data set (**G**, **H**) (#12)

**Fig. 2:**
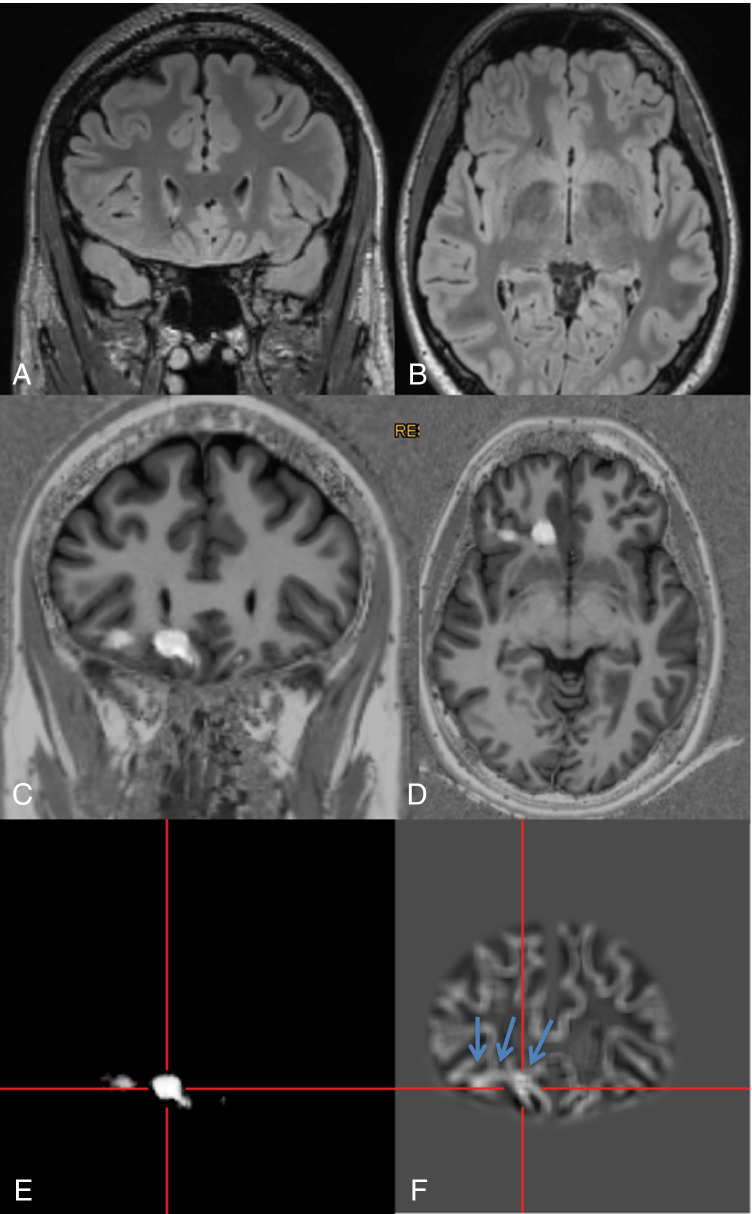
A 28-year-old man with hypomotor seizures and clonic seizures with versive head movement to the left side evolving to bilateral tonic–clonic seizures (#40). MRI with coronal (**A**) and axial reformations (**B**) of a 3D FLAIR sequence was considered to be normal. By scrolling through the co-registered MP2RAGE images (**C**, **D**), the lesion was detected “within a minute.” For co-registration, the ANN probability map (E) is used. The junction image as one of the input maps for the ANN highlights the blurring of the gray white matter junction as the most prevalent feature of FCD (**F**: arrows). Epileptogenicity of the lesion was confirmed by SEEG, the patient underwent surgery, and histopathology revealed a mild malformation of cortical development (mMCD)

**Fig. 3:**
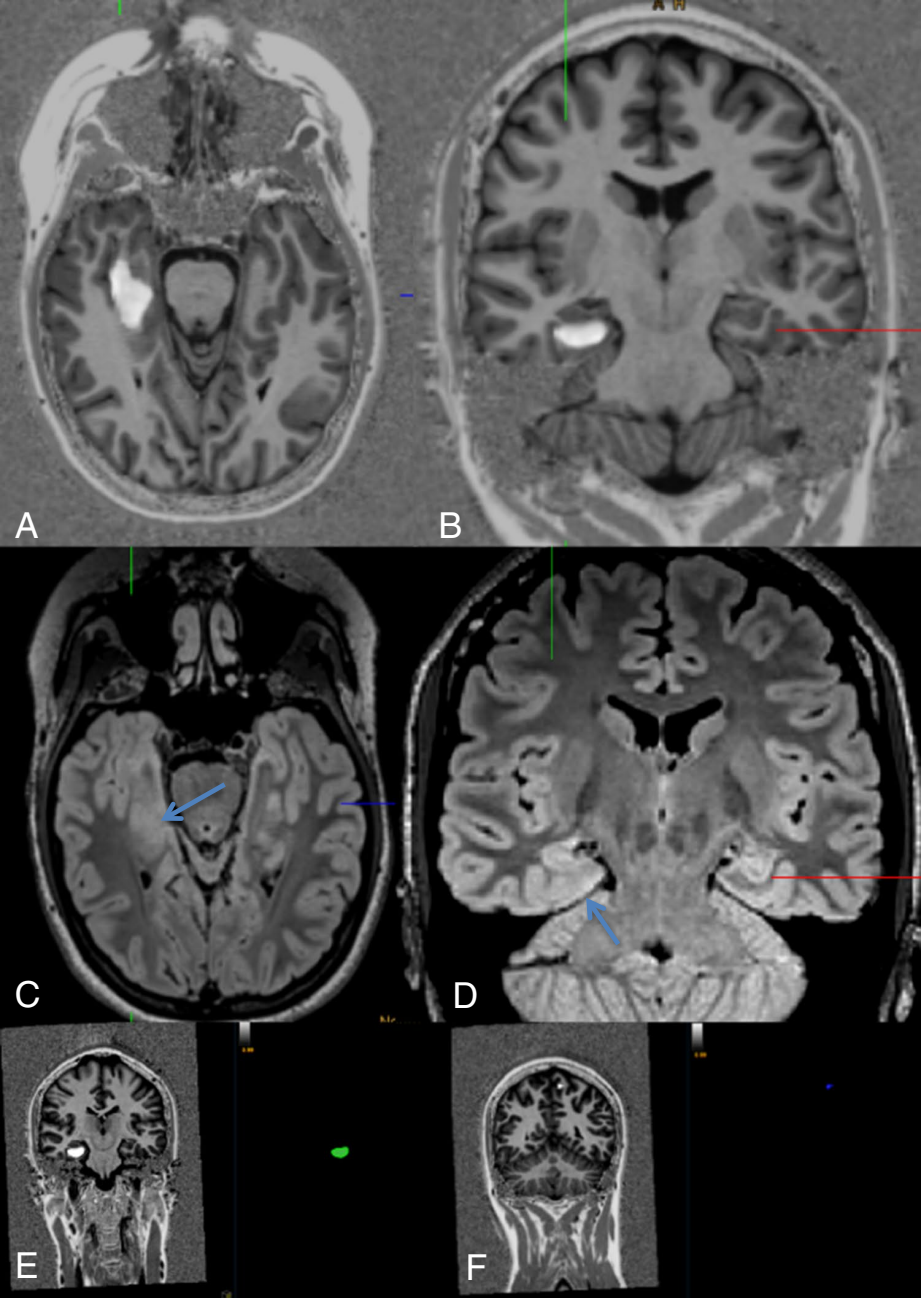
A 22-year-old woman with auditory, somatosensory, and visual auras evolving to bilateral tonic–clonic seizures. Postictal hemianopia to the left side (#22). Scrolling through the co-registered MP2RAGE data set led to the identification of an FCD of the right parahippocampal gyrus (**A**, **B**). Axial (**A**) and coronal FLAIR images showed a corresponding subtle blurring of the subcortical white matter (**C**, **D**). Intracranial stereo-EEG proved the lesion to be epileptogenic. Lesion volume was 1.6 ml (**E**) with a tiny false-positive lesion of the left superior frontal gyrus (0.02 ml) (**F**)

**Fig. 4:**
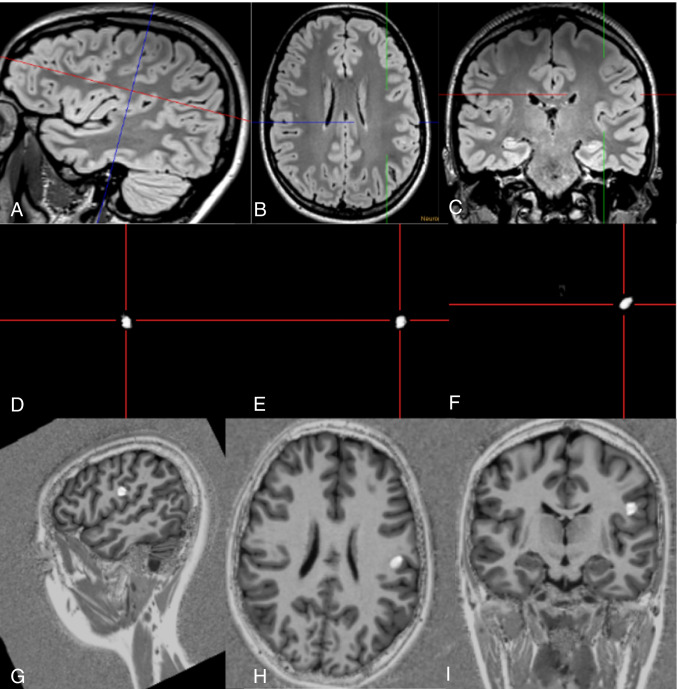
Subtle FCD of the left postcentral gyrus (#29). The lesion was detected on the 3D FLAIR sequence as it showed a subtle transmantle sign (**A**–**C**: crosshair). Junction images in sagittal (**D**), axial (**E**), and coronal (**F**) reformations showed a 0.33-ml large abnormality at the gray white matter junction. The co-registered MP2RAGE-ANN images confirmed the lesion (**G**–**I**). The patient underwent SEEG and subsequent surgery, confirming an FCD IIB

FCD are characterized by disordered cortical lamination with or without abnormal cell types. FCD are the most commonly resected epileptogenic lesions in children and the third most common lesions in adults [2]. Structural MRI abnormalities comprise an increased cortical thickness, blurring of the gray/white matter junction, a transmantle sign, and/or an abnormal gyral/sulcal pattern [3]. These abnormalities can be subtle but highlighted by voxel-based morphometric MRI analysis and comparison of results to a group of healthy controls. Using this approach, the morphometric analysis program (version of 2018; MAP18) utilizes MPRAGE data sets and generates morphometric maps in terms of junction, extension, and thickness images enhancing the visualization of abnormal blurring of the gray/white matter junction, abnormal extension of gray matter into deep white matter as well as an increased cortical thickness [4–6]. The sensitivity of FCD detection is higher by using MP2RAGE instead of MPRAGE sequences as potential FCD lesions are displayed with larger volumes and higher mean z-scores [7]. The MP2RAGE sequence is a MPRAGE sequence with two inversion pulses at 700 ms and 2500 ms, respectively. From the two images, a so-called unified image is calculated using the formula $\mathrm{MP}2\mathrm{RAGE}=\frac{\mathrm{contrast TI}1 \times \mathrm{ contrast TI}2}{{\mathrm{contrast T}1}^{2} + {\mathrm{contrast TI}2}^{2}}$. The MP2RAGE sequence produces images with a higher B_1_ homogeneity than the MPRAGE sequence and is therefore particularly suited for postprocessing [8, 9]. Another recent modification is the integration of an artificial neural network (ANN) in the postprocessing tool MAP18. This ANN was trained with morphometric and brain segmentation maps from MPRAGE sequences and generates FCD probability maps which reflect the voxel-wise probability for dysplastic tissue [10]. Here, although the ANN was trained with MPRAGE images, FCD probability maps were calculated from MP2RAGE images and presented as an overlay on the original MP2RAGE input image (cf. examples in Figs. 1, 2, 3, and 4).

We hypothesized that by presentation of original MR images together with co-registered maps resulting from morphometric MRI analysis, FCD can be detected very fast, i.e., “within one minute.” The aim of this prospective study was therefore to evaluate the feasibility and accuracy of such an approach.

## Methods

Within a 6-month period (1.9.2019–29.2.2020), consecutive patients with focal epilepsy syndromes suggested by semiology and/or EEG were included. Inclusion criteria were that patients had been considered MRI-negative so far and were studied with an epilepsy-dedicated protocol including a MP2RAGE sequence on a 3-T Prisma scanner (Siemens Healthineers, Erlangen, Germany) [7]. The study was in accordance with the 1964 Helsinki Declaration and its later amendments and approved by the local ethical committee.

### Postprocessing

The unified images of the MP2RAGE sequence were processed with SPM 12 (http://www.fil.ion.ucl.ac.uk/spm/) running in MATLAB R2014b (MathWorks, Natick, MA, USA). DICOM images were converted to NIfTI format; segmented into gray matter (GM), white matter (WM), and cerebrospinal fluid (CSF) maps; and normalized to the Montreal Neurological Institute (MNI) space. Using the MAP18 software, junction, extension, and thickness images were calculated as described before [4–6]. In addition, these morphometric maps were used as input for an artificial neural network (ANN) trained with MRI data of FCD patients and healthy controls as described elsewhere [10]. However, it should be noted that this ANN has been trained with MPRAGE data as FCD cases with MP2RAGE sequences required for such training are currently not available in sufficient quantity. The output of the ANN after classification of all voxels in the unified MP2RAGE image comprises a FCD probability map with values closer to 1 indicating voxels more likely to be dysplastic tissue and values closer to 0 representing non-dysplastic brain tissue or compartments outside of the brain. Finally, all results of MRI postprocessing (i.e., morphometric maps and FCD probability map) were inversely normalized (i.e., transferred to native space), co-registered, reconverted to DICOM format, and exported to the PACS system. The FCD probability map was displayed as an overlay in the original MP2RAGE image and could be screened very fast by scrolling through the co-registered data sets (Fig. 1).

### Evaluation

Epilepsy-dedicated MRI scans of 40 consecutive patients were evaluated by a senior neuroradiologist (> 20 years of experience) by visual inspection of the conventional MR images first, and then—for the “one minute” approach—together with the co-registered MRI postprocessing results. A FCD was diagnosed when the typical radiologic criteria [3] were clearly identifiable in the conventional MR images. A lesion was regarded as detected by the “one minute” approach (i.e., true positive, TP), if the results in the FCD probability map overlapped with the dysplastic lesion, thus allowing for a rapid detection. Furthermore, we differentiated between lesions that were already recognizable in the first screening of the conventional MRI images and those only detected in the “one minute” approach by means of co-registered postprocessing results. In order to calculate false positives (FP), we generated morphometric results including FCD probability maps of 36 healthy controls who had been studied to build up a normative database. Number and volumes of TP and FP clusters in the FCD probability map were measured using the imaging platform NORA (www.nora-imaging.org).

## Statistics

Descriptive statistics include age, gender, clinical findings/semiology, and location of lesions. The normal distribution of TP and FP FCD cluster volumes was checked by Shapiro–Wilk test. The Mann–Whitney U test was used to compare TP and FP FCD volumes. A receiver operating curve (ROC) was calculated to determine the optimal cut-off volume between TP and FP. All statistical analyses were performed using R statistics (R Core Team, https://www.R-project.org).

## Results

Out of 40 consecutive patients, 15 patients had FCD, three subcortical band heterotopia (SBH), one periventricular nodular heterotopia, and one focal polymicrogyria respectively. Twenty patients were MRI-negative (Table 1).

**Table 1:** Patient characteristics

ID	Age [years], sex	Clinical information	Lesion type (based on MR imaging)	Location	True positives: number; volume/ml	False positives: number; volume/ml	Presurgical work-up, treatment, histopathology, outcome
1	33, f	Suspected focal epilepsy	SBH	Bilateral parieto-occipital lobes	3; 43.43, 43.02, 0.1	0	Video EEG: left parieto-occipital epileptic discharges Drug therapy
2	33, m	Seizures with oral automatisms and aphasia	Polymicrogyria	R parietal lobe	0	0	Drug therapy
3	14, m	Seizures with auditive auras and manual automatisms	FCD	R inferior frontal gyrus	1; 0.13	3; 0.02, 0.01, 0.01	Video EEG: right fronto-temporal seizure origin, awaits further work-up (neuropsychology, FDG-PET)
4	30, m	Somatosensory and tonic-myoclonic seizures right face, grimassing, choking	FCD	L inferior frontal gyrus	1; 0.29	6; 0.33, 0.06, 0.02, 0.01, 0.01, 0.01	Language fMRI: left-sided lateralization. Subdural grid covering the inferior left frontal gyrus scheduled
5	36, m	Tonic to bilateral tonic–clonic seizures	FCD	R middle frontal gyrus	4; 13.12, 1.1, 0.12, 0.04	6; 0.25, 0.18, 0.04, 0.02, 0.02, 0.01	SEEG recommended after proving drug resistance
6	36, f	R-sided TLE of unclear origin with gustatory auras, vegetative and impaired awareness seizures	None	n.a	0	1; 0.08	Further drug resistance testing
7	16, f	Nocturnal hyperkinetic seizures	FCD	R superior frontal gyrus	1; 0.24	5; 0.32, 0.12, 0.03, 0.01, 0.01	Seizure free with drug therapy
8	24, m	focal impaired awareness to bilateral tonic–clonic seizures	FCD	L cingulate gyrus + middle frontal gyrus	7; 7.33, 1.73, 0.53, 0.1, 0.08, 0.07, 0.05	2; 0.63, 0.04	Vagus nerve stimulation
9	15, f	Dyscognitive and bilateral tonic–clonic seizures	None	n.a	0	7; 0.18, 0.04, 0.05, 0.2, 0.01, 0.06, 0.02	Seizure free with drug therapy
10	30, m	Focal impaired awareness to bilateral tonic–clonic seizures	None	n.a	0	0	Drug therapy
11	51, m	No epilepsy, PNES	None	n.a	0	0	No epilepsy therapy
12	37, m	Tonic and bilateral tonic–clonic seizures with accentuation on the left side	FCD	R superior frontal gyrus right	1; 0.52	0	Surgery performed HP: FCD IIB Postsurgical outcome (3 months) pending
13	18, m	R parietal lobe epilepsy	None	n.a	0	0	Drug therapy
14	59, m	PNES	None	n.a	0	0	Predisposition to generalized epilepsy with generalized epileptic discharges
15	33, f	Focal seizures right arm and bilateral tonic–clonic seizures	FCD	L inferior frontal gyrus	0	1, 0.08	Awaits further presurgical work-up
16	20, f	TLE with Déjà-vu auras and bilateral tonic–clonic seizures	None	n.a	0	0	Seizure free with drug therapy
17	31, f	TLE with focal seizures	None	n.a	0	1; 0.07	Seizure free with drug therapy
18	25, f	Sensory seizures with automatisms and receptive aphasia	None	n.a	0	1; 0.04	Drug therapy
19	21, m	Clonic seizures right hand	None	n.a	0	2; 0.29, 0.02	Subdural EEG with likely focus within the primary hand region
20	38, m	Single bilateral tonic–clonic seizure, PNES	None	n.a	0	2; 0.08, 0.03	Seizure free with drug therapy
21	31, f	Focal impaired awareness seizures with oral automatisms	SBH	Bilateral parieto-occipital lobes	5; 30.47, 25.15, 0.22, 0.16, 0.04	0	Drug therapy
22	22, f	Auditory, sensory (left arm) and visual (upper left quadrant) auras with bilateral tonic–clonic seizures	FCD	R parahippocampal gyrus	2; 1.5, 0.1	7; 0.26, 0.11, 0.03, 0.02, 0.01, 0.01, 0.01	SEEG and surgery performed HP: mild malformation type II Seizure free after 3 months
23	17, m	Apractic seizures and motor seizures involving the right arm and bilateral tonic–clonic seizures	None	n.a	0	2; 0.22, 0.2	SEEG performed: extended R-temporo-parieto-occipital epileptogenic area
24	40, f	Bilateral tonic to bilateral tonic–clonic seizures	None	n.a	0	2; 0.12, 0.01	No circumscribed hypothesis for seizure origin
25	11, m	Tonic seizures left arm with loss of tone in the trunc	FCD	R cingulate gyrus	1.11	19 (total volume 3.13)	Surgery performed HP: FCD IIB Seizure free 12-month post-surgery
26	13, f	Epigastric auras to bilateral tonic–clonic seizures	SBH	Bilateral parieto-occipital lobes	7; 4.32, 0.11, 2.79, 0.02, 0.58, 0.5, 1.39	2; 0.05, 0.03	Drug therapy
27	49, f	TLE left with acoustic auras and focal impaired awareness to bilateral tonic–clonic seizures, PNES	None	n.a	0	23 (total volume 3.11)	Seizure free with drug therapy, PNES persistent
28	27, m	TLE left with epigastric and gustatory auras and focal impaired awareness to bilateral tonic–clonic seizures	None	n.a	0	0	Drug therapy
29	18, f	Tonic seizures with versive head movement to right to bilateral tonic–clonic-seizures	FCD	L postcentral gyrus	0.33	0	SEEG and surgery performed HP: FCD IIB
30	7, m	No epilepsy, autism spectrum disorder	None	n.a	0	11 (total volume 1.58)	None
31	17, f	Focal epilepsy of unknown origin	None	n.a	0	1; 0.01	Drug therapy
32	26, f	L-sided temporo-parieto-occipital epilepsy with unspecific auras, vegetative and aphasic seizures	FCD	L occipital and inferior parietal lobule	2; 1.86, 0.29	1; 0.1	Additional long-term video EEG
33	15, m	Acoustic auras and hyperkinetic impaired awareness seizures	FCD	L superior temporal gyrus	2; 1.3, 1.25	4; 0.06, 0.04, 0.01, 0.01	Surgery performed HP: FCD IIB Seizure free 6-month post-surgery
34	21, m	TLE left with hyperkinetic impaired awareness and bilateral tonic–clonic seizures	Gray/white matter blurring	L temporal pole	0.45	1; 0.05	Seizure free with drug therapy
35	42, f	Bilateral tonic–clonic seizures	None	n.a	0	4; 0.09, 0.04, 0.01, 0.01	Drug therapy
36	12, f	R-sided TLE with hyperkinetic impaired awareness to bilateral tonic–clonic seizures	FCD	L superior temporal gyrus	0.87	2; 0.03, 0.02	Seizure free with drug therapy
37	25, m	L-sided TLE with hyperkinetic and bilateral tonic–clonic seizures	FCD	R middle frontal gyrus	0.86	4; 8.29, 1.78, 1.5, 0.02	SEEG not possible because of ambulatory hyperkinetic seizures
38	35, m	R-hemispheric focal seizures with presumed temporoposterior/occipital origin	None	n.a	0	2; 0.17, 0.02	Drug therapy
39	16, m	R-sided TLE	Periventricular nodular heterotopia	R temporal horn	0	4; 0.2, 0.1, 0.23, 0.03	Drug therapy
40	28, m	Hypomotor seizures and clonic seizures with versive head movement to left	FCD	R frontoorbital	1.73	2; 0.01, 0.01	Confirmed with SEEG, surgery performed HP: mMCD

*f* female, *HP* histopathology, *L* left, *m* male, *n.a.* not applicable, *PNES* psychogenic non-epileptic seizures, *R* right, *SBH* subcortical band heterotopia, *SEEG* stereo electroencephalography, *TLE* temporal lobe epilepsy, *mMCD* mild malformation of cortical development

Four FCD (## 7, 8, 22, 40; i.e., 27% of FCD) and one SBH (#25; i.e., 33% of SBH) were only detected by postprocessing using “within a minute” approach (Figs. 2, 3). One FCD with a subtle transmantle sign was missed with the “within a minute” approach and only visible in the junction image of the MAP (#15). The focal polymicrogyria (#2) was not visualized with the “within a minute” approach.

In another two patients, the extent of the FCD displayed by postprocessing was visually larger than in the conventional MR images (##32, 33).

FPs were found in 29 patients: 11 patients had no FP, 16 patients had one or two FP clusters with a volume of 0.01–0.63 ml, and 13 patients more than two, often scattered FP clusters (volumes of 0.01–8.29 ml), respectively.

Median lesion volume of true positive (TP) clusters was 0.53 ml (0.04–13.1 ml; IQR 1.17) compared to 0.04 ml (0.01–8.29 ml; IQR 0.10) of the FP clusters (*p* < 0.001).

The AUC of the receiver operating curve was 0.851 with a cut-off volume of 0.05 ml best indicating a true dysplastic lesion (*n* = 15).

With respect to FPs in healthy controls, 14 of 36 had no FP, 11 subjects had singular FP clusters, three patients had two, and eight patients more than two FP clusters, respectively. The median volume of FP was 0.09 ml (0.01–4.29 ml, IQR 0.28).

## Discussion

FCD can be detected “within a minute” when MP2RAGE data sets are postprocessed by morphometric analysis and then presented together with morphometric results in the PACS, thus allowing for a very fast screening by scrolling through the co-registered images. In the present study, this approach helped to detect four FCD and one subcortical band heterotopia which otherwise would have been overlooked. In another two patients, the lesions were larger in the postprocessed compared to the conventional images which encourages the reader to consider it a lesion and not a FP. Yet, there were also FP clusters in the FCD probability maps, both in patients and in healthy controls. However, volumes of true dysplastic lesions were significantly larger than FP clusters, at least in our study population.

According to the ROC analysis of 15 FCD, a threshold size of 0.05 ml differentiated best between FCD and FP findings in our study. The number of underlying FCD cases is too small to make a definite recommendation for a threshold value here. It is probably also not possible to determine a fixed lower limit for the size of FCDs, as they may become arbitrarily small. In any case, if a lesion is suspicious according to postprocessing, it is essential to check whether the typical criteria of FCD can also be identified in the conventional MR images.

One limitation of our study also offers hope for the future: for the generation of the FCD probability maps, we used the artificial neural network (ANN) currently available in MAP18, which was trained on MPRAGE data sets. A new training using MP2RAGE data for this study was not possible so far due to the low numbers of FCD with MP2RAGE sequence. In this respect, the false-positive findings could also be partly a consequence of this still insufficient adaptation. Future training with MP2RAGE data might reduce the number of FP and increase both sensitivity and specificity for FCD detection. For FCD detection during a presurgical work-up, sensitivity is more important than specificity or the number of FPs. An undetected FCD may prevent the patient from further work-up, but a putative FCD would not be operated without proving its epileptogenicity using clinical and electrophysiological data. Therefore, a high sensitivity is more crucial in this clinical situation than a high specificity.

The morphometric analysis program (MAP) is integrated in standard presurgical workflows of over 60 epilepsy centers in 22 different countries [10]. It has independently been validated for its clinical benefits against expert neuroradiological assessments [11–13] with potential impact on further, also invasive and presurgical patient management [14]. The resulting morphometric maps as well as the FCD probability map resulting from the recently integrated ANN [6] can be easily inversely normalized back to the native space within the program and directly used for stereotactic and/or neuronavigation procedures. The program typically processes MPRAGE sequences with isotropic 1mm^3^ large voxels; the MPRAGE sequence itself is a fundamental part of epilepsy imaging protocols [15–17]. However, House and co-workers found higher mean z-scores of FCD when processing 3D T2-weighted instead of MPRAGE data sets [18]. We recently showed that not only the mean z-scores but also the volumes of FCD are larger when processing MP2RAGE instead of MPRAGE data sets. Especially, the higher volume of FCD facilitated the separation from FP when scrolling through the MP2RAGE data sets [7]. By displaying larger and brighter lesions, the MP2RAGE sequence increases the diagnostic confidence which may have an impact on the decision to proceed with invasive EEG recordings or epilepsy surgery. The higher diagnostic yield of the MP2RAGE sequence likely results from the intrinsic correction of B1 inhomogeneities which is achieved by combining two MPRAGE data sets acquired interleaved at different inversion times [7–9]. The drawback is a longer acquisition time (≈ 8 versus 4 min) and the higher risk for movement artifacts.

The major limitation of this study is that only six of 15 patients with FCD have been operated so far (## 12, 22, 25, 29, 33, 40). Four of those underwent stereo-electroencephalographic (SEEG) recordings which clearly showed ictal EEG activity within and around the lesions. Here, the “one minute” approach led to extended electrode implantations proving the accuracy of the findings with SEEG recordings (#22, Fig. 3).

In conclusion, by postprocessing and displaying a MP2RAGE sequence and the co-registration output map in the PACS, subtle or even unvisible FCD can be detected “within a minute.” Fully automated MRI analyses have the potential to easily identify FCD and to contribute to overcome the underutilization of epilepsy surgery and prolonged latencies for referral to epilepsy centers.

## Data Availability

On reasonable request to corresponding author, the underlying data can be accessed.

## Abbreviations

FCD: Focal cortical dysplasia
SBH: Subcortical band heterotopias
MAP: Morphometric analysis program
ANN: Artificial neural network
TP: True positives
FP: False positives
PACS: Picture archiving communication system

## Code availability

MAP18 and re-integration into a PACS are based on the freely available Statistical Parametric Mapping toolbox (SPM12, Wellcome Trust Centre for Human Neuroimaging, London, UK). MAP18 is administered by Huppertz HJ, Zurich, Switzerland.
